# Sex and gender role differences on stress, depression, and anxiety symptoms in response to the COVID-19 pandemic over time

**DOI:** 10.3389/fpsyg.2023.1166154

**Published:** 2023-05-03

**Authors:** Maryse Arcand, Alexe Bilodeau-Houle, Robert-Paul Juster, Marie-France Marin

**Affiliations:** ^1^Département de Psychiatrie et d'Addictologie, Faculté de Médecine, Université de Montréal, Montréal, QC, Canada; ^2^Centre de Recherche de l'Institut Universitaire en Santé Mentale de Montréal, Montréal, QC, Canada; ^3^Département de Psychologie, Faculté des Sciences Humaines, Université du Québec à Montréal, Montréal, QC, Canada

**Keywords:** stress, anxiety, depression, gender role, sex differences, long-term stressful event

## Abstract

**Introduction:**

Stress, depression, and anxiety symptoms have been reported during the pandemic, with important inter-individual differences. Past cross-sectional studies have found that sex and gender roles may contribute to the modulation of one's vulnerability to develop such symptoms. This longitudinal study aimed to examine the interaction of sex and psychological gender roles on stress, depression, and anxiety symptoms in adults during the COVID-19 pandemic.

**Methods:**

Following the confinement measures in March 2020 in Montreal, stress, depression, and anxiety symptoms were assessed every 3 months (from June 2020 to March 2021) with the Depression, Anxiety and Stress Scale among 103 females and 50 males. Femininity and masculinity scores were assessed with the Bem Sex Role Inventory before the pandemic and were added as predictors along with time, sex, and the interactions between these variables using linear mixed models.

**Results:**

We observed similar levels of depressive symptoms between males and females, but higher levels of stress and anxious symptoms in females. No effects of sex and gender roles on depressive symptoms were found. For stress and anxiety, an interaction between time, femininity, and sex was found. At the beginning of the pandemic, females with high femininity had more stress symptoms than males with high femininity, whereas females with low femininity had more anxiety symptoms 1 year after the confinement measures compared to males with low femininity.

**Discussion:**

These findings suggest that sex differences and psychological gender roles contribute to heterogeneous patterns of stress and anxiety symptoms over time in response to the COVID-19 pandemic.

## 1. Introduction

The health crisis provoked by the COVID-19 pandemic has had significant repercussions on the mental health of individuals worldwide. Indeed, several studies have shown a high prevalence of stress, anxiety, and depressive symptoms in the general population (Luo et al., 2020; Salari et al., 2020; Xiong et al., 2020). These findings have been especially present among females (Findlay et al., 2020; Newby et al., 2020; Özdin and Bayrak Özdin, 2020; Xiong et al., 2020). Meta-analyses of cross-sectional studies reported prevalence rates in these samples of up to 29.6% for stress symptoms, between 14.6% and 48.3% for depressive symptoms, and between 6.3% and 50.9% for anxiety symptoms for people living mainly in Asia, as well as those living in Europe, the Middle East, United States, and Latin America (Luo et al., 2020; Salari et al., 2020; Xiong et al., 2020). In Canada, one study reported a deterioration of mental health in the general population during the first wave of the pandemic, characterized by an increase in anxiety and depressive symptoms (Robillard et al., 2021). The results of the latter study also showed that an increase in perceived stress during the pandemic was associated with an exacerbation of anxiety and depressive symptoms.

Given that most studies have used cross-sectional designs and the heterogeneity of the epidemiological evolution across the globe, the long-term mental health impact of the COVID-19 pandemic and confinement measures have been difficult to pinpoint. While several studies have reported an increase in anxiety and depressive symptoms at the onset of the health crisis relative to pre-pandemic symptoms, one study reported a decrease in symptoms mid-pandemic (Robinson et al., 2021), while another reported maintenance of symptoms (Daly et al., 2020). Although acute stress responses are adaptive and healthy (McEwen, 1998), chronic stress can lead to the dysregulation of the stress system and contribute to the development of psychopathologies such as anxiety and depressive disorders (McGonagle and Kessler, 1990; Staufenbiel et al., 2013). This highlights the need to better understand individual differences in resilience and vulnerability associated with the evolution of psychiatric symptoms over time.

Anxiety and depression are the most common psychopathologies in society and are highly comorbid. Indeed, a global study conducted by Kessler et al. (2015) showed that 45.7% of people who suffered from a depressive disorder in their lifetime had also suffered from one or more anxiety disorders. Sex differences for anxiety and depression are also observed starting from puberty onwards, with a higher vulnerability among females than males (Altemus et al., 2014; Baxter et al., 2014; Remes et al., 2016; Lim et al., 2018). Beyond these sex differences, studies have shown that including psychosocial variables in research (e.g., psychological gender roles) allows for a more profound understanding of these symptoms in individuals (Lengua and Stormshak, 2000; Palapattu et al., 2006).

As a concept distinct from birth-assigned sex, psychological gender roles are defined by the adherence of males and females to socially transmitted stereotypical characteristics associated with femininity and masculinity (Bem, 1981). Indeed, humans are socialized to incorporate personality traits, roles, characteristics, and attitudes that correspond to one's birth-assigned sex (Bem, 1981). Some argue that mental health is strongly modulated by psychological gender roles, as studies have demonstrated the influence of these roles on self-esteem, emotional regulation, psychological adjustment, and coping strategies (Bem, 1981; Jones et al., 2016). Despite criticisms regarding the year in which the instrument was created (Donnelly and Twenge, 2017), the Bem Sex-Role Inventory (Bem, 1974) is one of the most widely used questionnaires to assess psychological feminine and masculine traits (Beere, 1990; Hoffman and Borders, 2001). This instrument was developed to measure typically masculine desirable or instrumental traits, which refers to self-oriented characteristics and the achievement of personal goals (i.e., strong, assertive personality, willingness to take risks, defending one's beliefs). In addition, the instrument was developed to measure typically feminine desirable or expressive traits, which refers to characteristics oriented around connecting with others (i.e., understanding, gentle, warm, sensitive to the needs of others; Bem, 1974).

Rather than being conceptualized as two extremes on one continuum, Bem argued that femininity and masculinity are two independent continuums (Bem, 1974; Spence et al., 1975). In the literature, gender roles have been studied using either categorical or continuous methods. Originally proposed by Bem, the categorical method allows for the creation of groups by considering the level of endorsement of feminine and masculine traits. These groups are defined as follows: feminine (high in femininity), masculine (high in masculinity), androgynous (both high in femininity and high in masculinity), and undifferentiated (both low in femininity and low in masculinity; Bem, 1974). A large body of literature based on this method has shown that an androgynous gender role is associated with better psychological adjustment (Bem, 1974), fewer stress symptoms (Littlefield, 2004), social anxiety (Goodman and Kantor, 1983), and depressive symptoms (Cheng, 2005; Juster et al., 2016; Vafaei et al., 2016). In contrast, undifferentiated gender roles have been associated with poorer mental health (Bem, 1977), higher levels of social anxiety (Goodman and Kantor, 1983), and depression (Flett et al., 2009; Szpitalak and Prochwicz, 2013).

However, as the creation of groups using the categorical method is based on the median of masculinity and femininity of the sample, an individual's gender role is dependent on the study's sample and could easily vary across studies. Consequently, this method significantly impacts the external validity of the research being conducted (Sedney, 1981). Moreover, by classifying individuals as feminine or masculine gender-typed, the use of this method may lead to an under and overestimation of the contribution of the non-dominant and dominant gender roles, respectively (Johnson et al., 2006). Alternatively, the continuous method measures the distinct contributions of femininity and masculinity along continuums without impacting the generalization of the results.

To date, few studies have investigated the impact of categorical gender roles on stress symptoms and no research has explored the impact of gender roles (conceptualized as a continuous method) on stress symptoms specifically. Moreover, past studies exploring this research question were conducted using female-only samples. The latter presents an important limitation for our understanding of the interaction between gender roles and sex differences.

Regarding depressive symptoms, studies have shown that greater identification with masculine traits is associated with lower levels of depression (Feather, 1985; Whitley, 1985; Nezu and Nezu, 1987; Stoppard and Paisley, 1987; Grimmell and Stern, 1992; Thornton and Leo, 1992; Waelde et al., 1994; Bromberger and Matthews, 1996; Lengua and Stormshak, 2000; Gibson et al., 2016; Arcand et al., 2020). For the association between depression and feminine traits, mixed and null results have been reported in the literature (Tinsley et al., 1984; Feather, 1985; Whitley, 1985; Stoppard and Paisley, 1987; Grimmell and Stern, 1992; Waelde et al., 1994; Cheng, 1999; Lengua and Stormshak, 2000; Stoyanova and Hope, 2012; Gibson et al., 2016; Arcand et al., 2020). For anxiety symptoms, studies have reported that masculinity is negatively associated with both anxiety (Nezu and Nezu, 1987; Eisler et al., 1988; Grimmell and Stern, 1992; Kleinplatz et al., 1992; Thornton and Leo, 1992; Stoyanova and Hope, 2012) and social anxiety (Moscovitch et al., 2005; Johnson et al., 2006). On the other hand, femininity has been positively correlated with anxiety symptoms (Palapattu et al., 2006; Blashill and Hughes, 2009; Arcand et al., 2020), social anxiety (Johnson et al., 2006), and phobic reactions (Blashill and Hughes, 2009).

The aforementioned studies have largely examined the global impact of gender roles on symptoms irrespective of birth-assigned sex. However, some studies have investigated whether sex moderates the contribution of psychological gender roles. For depressive symptoms, mixed results have been reported for both femininity and masculinity, with some studies finding associations in females (Lengua and Stormshak, 2000) and males (Szpitalak and Prochwicz, 2013; Gibson et al., 2016), while others found no sex differences at all (Feather, 1985; Waelde et al., 1994; Gibson et al., 2016; Vafaei et al., 2016). For stress and anxiety symptoms, an interaction between sex and gender roles has yet to be found (Nezu and Nezu, 1987; Moscovitch et al., 2005).

Of note, past studies have largely used cross-sectional designs. Indeed, only a few longitudinal studies have examined the effect of gender roles on depressive symptoms. A study by Cheng (1999) conducted on healthy young adults across two time points (with a 6-month interval) found that masculinity was negatively associated with depressive symptoms. These findings suggest that masculinity stably predicts fewer stress symptoms over time (Cheng, 1999). Inversely, in a sample of postmenopausal females, few masculine traits were associated with an increase in depressive symptoms measured 3 years later (Bromberger and Matthews, 1996). As measured across four time points over a 15-year period, only one study has reported no effect of gender roles on depressive symptoms in healthy adults (Wilhlem et al., 1998). To the best of our knowledge, no longitudinal study has explored the effects of gender roles on stress and anxiety symptoms. In addition, studies that have measured the impact of gender roles on symptoms of stress, depression, and anxiety have focused on general symptoms without considering a specific stressor and its temporality. Symptoms of stress, anxiety, and depression have been exacerbated and maintained over time as a result of the COVID-19 pandemic. Furthermore, the entirety of the general population was exposed to the same prolonged stressor (i.e., the pandemic). For this reason, it is important to better understand the effect of gender roles and sex differences to identify the risk factors associated with the development and maintenance of these symptoms.

The goal of our study was to examine the effects of sex and gender roles, as well as the joint effect of sex and gender roles, on stress, depression, and anxiety symptoms during the COVID-19 pandemic. Based on the literature, we formulated the following hypotheses: (1) females will exhibit more symptoms of stress, depression, and anxiety than males at the four time points; (2) higher masculinity will be associated with lower symptoms of stress, anxiety, and depression over the course of a year; (3) femininity will be associated with more anxiety symptoms. Considering the lack of data regarding the effect of gender roles on stress symptoms and mixed results regarding the impact of femininity on depression, we did not formulate a directional hypothesis for the association between gender roles and stress symptoms, nor for the association between femininity and depression. In addition, given the lack of data on sex differences concerning gender roles, no directional hypothesis was formed for the interactions between sex and gender roles.

## 2. Method and measure

### 2.1. Sample and study design

Across three different studies conducted in our laboratory, 160 participants between the ages of 19 and 54 years old were recruited via social media and bulletin boards in the Montreal area between July 2017 and March 2020. Upon recruitment, a telephone screening interview was conducted to ensure that participants were physically and mentally healthy and were not taking any medications for mental illnesses. All participants provided their written consent and took part in a laboratory-based experiment involving self-report questionnaires, as well as cognitive and emotional tasks. As a part of their participation in one of these three studies, gender roles were measured in all participants (T0).

**Figure 1:**
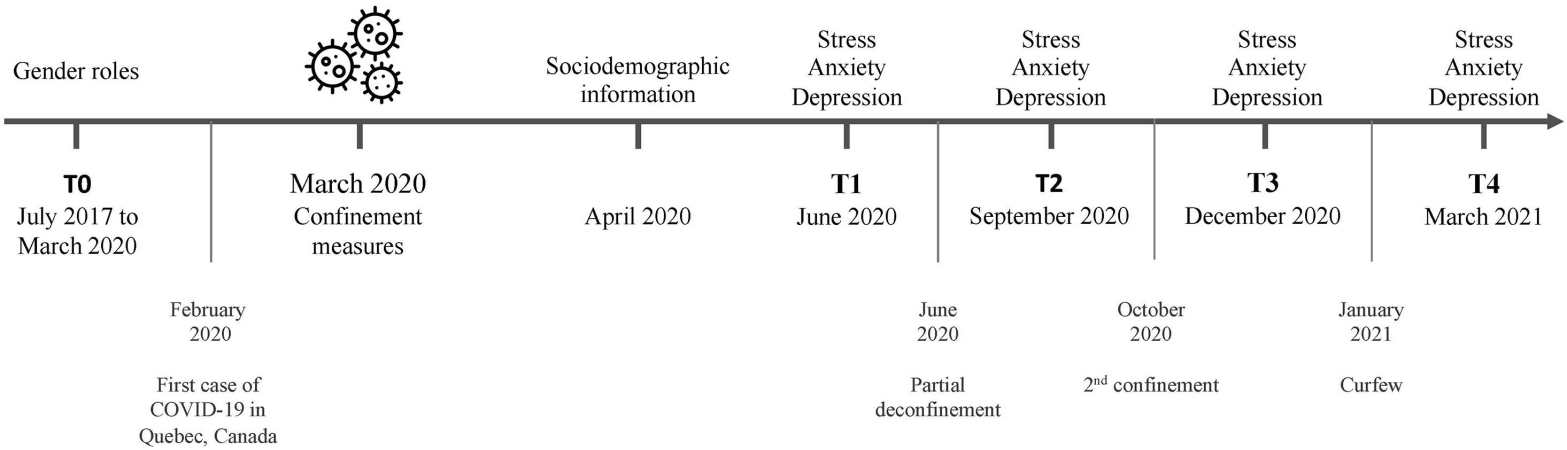
Timeline overview. Symptoms of stress, depression, and anxiety were measured at T1 in June 2020 (+3 months), T2 in September 2020 (+6 months), T3 in December 2020 (+9 months), and T4 in March 2021 (+12 months).

The first case of COVID-19 was reported in Quebec in February 2020. In March 2020, all non-essential facilities and services were disrupted (i.e., closure of schools, daycare centers, workplaces, restaurants, and entertainment venues) and a provincial confinement was declared to limit the spread of the virus. Thereafter, the evolution of the confinement measures varied as a function of the epidemiological situation, see Figure 1 for a timeline overview. All participants recruited before the start of the pandemic (*n* = 160; T0) were re-contacted. In total,159 individuals agreed to take part in the study in May 2020. Gender role data was not available for six participants and thus, they were removed from the current analyses. Therefore, the final sample for the current study included data from 103 females and 50 males. After obtaining consent, sociodemographic data were collected, including measures of self-reported sex (female/male or prefer not to answer). Afterwards, symptoms of stress, depression, and anxiety were measured online at four post-confinement time points: T1 in June 2020 (+3 months), T2 in September 2020 (+6 months), T3 in December 2020 (+9 months), and T4 in March 2021 (+12 months). All self-report data were collected via Qualtrics, a highly secured online platform. For each time point, a personalized URL was sent to each participant by email. From T0, the attrition rate for each time point was as follows: T1 = 2.0 %, T2 = 7.2 %, T3 = 13.7 %, and T4 = 9.8 %. At the end of the study, participants received financial compensation that was proportional to their involvement in the study (ranging from $10 to $50). Ethics approval was obtained from the institutional review board of the *CIUSSS-de-l'Est-de-l'Île-de-Montréal*. This study was conducted in conformity with the Declaration of Helsinki.

### 2.2. Measures

#### 2.2.1. Gender roles

The 30-item short form of the Bem Sex-Role Inventory (Bem, 1974) was used to assess psychological gender roles. This questionnaire is composed of subscales focusing on feminine (e.g., “sensitive to the needs of others”) and masculine (e.g., “independent”) stereotyped traits. The questionnaire had 10 items for each of the following traits: feminine, masculine, and neutral traits (the 10 neutral items were not used for the current manuscript). Participants were asked to rate each item using a Likert scale from 1 (never or almost never true) to 7 (always or almost always true), yielding a score between 10 and 70 for each subscale (i.e., femininity and masculinity). In this study, Cronbach's alphas for the masculine and feminine subscales of the French version of the questionnaire are 0.79 and 0.74, respectively.

#### 2.2.2. Depression, anxiety, and stress symptoms

The 21-item Depression, Anxiety, and Stress Scale (DASS-21; Lovibond and Lovibond, 1995) is composed of three subscales which feature seven items evaluating each of the following: depression, anxiety, and stress. Participants had to rate each item using a Likert scale from 0 (does not apply to me at all) to 3 (applies to me entirely or the vast majority of the time). The items evaluating each subscale were summed and multiplied by two, yielding a score between 0 and 42. We used the French version of the questionnaire that was developed by Donald Martin's team from the University of Ottawa (available on the Internet at: French translation of the Donald, 2012). In our sample, we found good internal consistency such that Cronbach's alpha coefficients ranged from 0.8 to 0.83 depending on the subscale.

### 2.3. Statistical analyses

#### 2.3.1. Preliminary analyses

To determine whether feminine traits, masculine traits, and age differed between males and females, we conducted independent samples *t*-tests with sex as the between-group variable. Then, we tested if symptoms of stress, depression, and anxiety at the four time points were correlated with potential covariates identified in the literature. Examples of these covariates include status (worker, student), level of education, income, having children, residence type (apartment, house), demographic location (urban, rural), and the number of people living in the household during the COVID-19 pandemic (Arcand et al., 2020; Özdin and Bayrak Özdin, 2020; Vindegaard and Benros, 2020).

#### 2.3.2. Principal analyses

To assess the impact of sex and gender roles, as well as the joint effect of these variables on symptoms of stress, depression, and anxiety, we conducted linear mixed-effects models. Given the longitudinal nature of this study, these analyses allowed us to control for the dependency of the data that may occur when collecting repeated measures. Statistical analyses were performed using RStudio software, version 1.4.1106 for macOS. Distinct models were conducted for stress, depression, and anxiety symptoms. First, to examine the main effects, we entered time, sex, femininity, and masculinity as fixed effects into the model. As symptoms were nested within participants, random intercepts were included. Second, to assess the interaction between sex and gender roles, as well as the interaction between sex and femininity scores/masculinity scores, these interaction terms were added to the model as fixed effects. Residual plots were visually inspected for normality and homoscedasticity and did not reveal any obvious deviations for both statistical assumptions. We used the maximum likelihood estimate with random assumptions for all linear mixed-effects models. This estimate provided unbiased estimates and valid inferences in the case of missing data. Significant interactions were further decomposed using *post hoc* contrast comparison tests.

## 3. Results

### 3.1. Preliminary analyses

Table 1 features sample characteristics according to sex. The independent *t*-tests revealed no difference between males and females for age [*t*_(151)_ = 0.423, *p* = 0.673] and feminine traits [*t*_(139)_ = −1.202, *p* = 0.231]. A trend effect was found for masculine traits [*t*_(139)_ = 1.878, *p* = 0.062], where males (M = 4.68, SE = 0.11) tended to have more masculine traits than females (M = 4.41, SE = 0.09). The correlation matrix showed no effect of status (worker, student), education level, income, having children, residence type (apartment, house), demographic location (urban, rural), and the number of people living in the household on stress, depression, and anxiety symptoms at all four time points. Therefore, none of these variables were used as covariates in the main analyses.

**Table 1:** Characteristics of the sample.

	**Full sample**	**Females**	**Males**
	***N***		
	153	103	50
T1	150	102	48
T2	142	96	46
T3	132	92	40
T4	138	95	43
**Mean (SEM)**			
Age	34.00 (0.80)	32.21 (0.93)	37.65 (1.40)
Femininity	5.73 (0.06)	5.78 (0.07)	5.64 (0.10)
Masculinity	4.50 (0.07)	4.41 (0.09)	4.68 (0.10)
**Anxiety**			
T1	4.75 (0.45)	5.29 (0.55)	3.65 (0.74)
T2	4.09 (0.44)	5.16 (0.59)	1.96 (0.46)
T3	4.26 (0.43)	5.48 (0.58)	1.76 (0.35)
T4	4.91 (0.46)	5.35 (0.57)	3.95 (0.74)
**Stress**			
T1	10.79 (0.61)	11.73 (0.77)	8.86 (0.94)
T2	10.78 (0.66)	12.22 (0.86)	7.92 (0.83)
T3	11.08 (0.70)	12.81 (0.87)	7.57 (0.98)
T4	11.94 (0.75)	13.03 (0.93)	9.53 (1.15)
**Depression**			
T1	6.37 (0.57)	6.69 (0.76)	5.73 (0.80)
T2	5.46 (0.51)	6.14 (0.70)	4.12 (0.57)
T3	5.85 (0.53)	6.67 (0.70)	4.16 (0.69)
T4	7.14 (0.62)	7.43 (0.79)	6.51 (0.97)

Standard errors of the mean (SEM) are presented in parentheses.

### 3.2. Main analyses for stress

**Figure 2:**
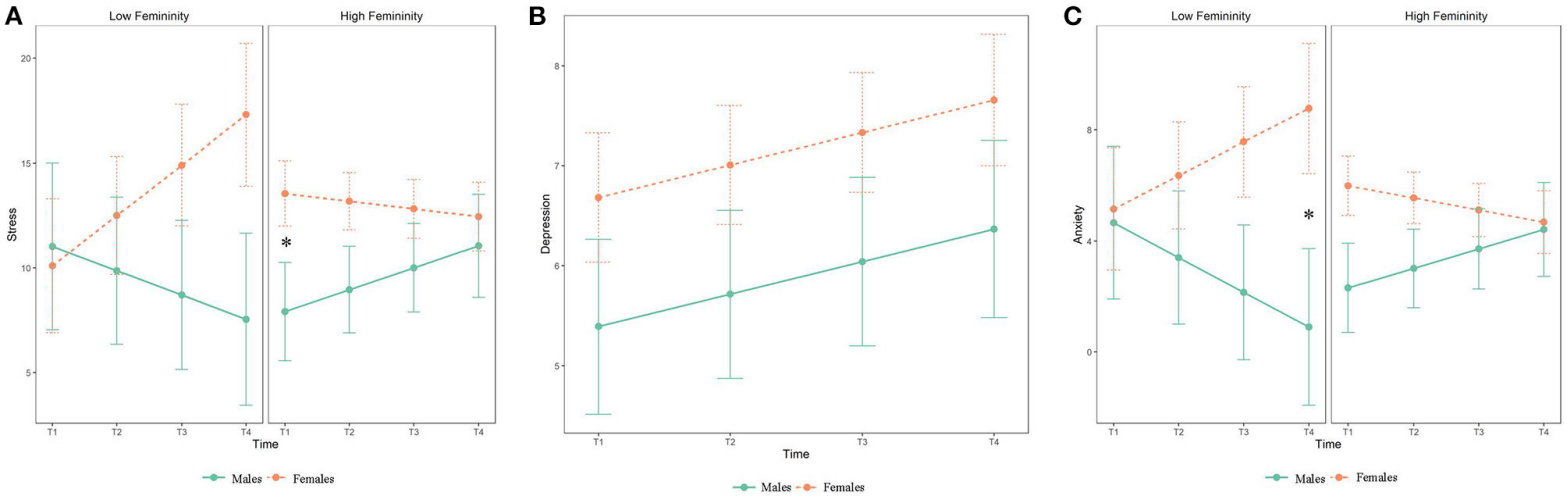
**(A, C)** Depict the joint effect of sex and gender roles on stress symptoms and anxiety symptoms as a function of time. **(B)** Depicts depression levels as a function of sex and the different measurement times. Low femininity refers to individuals with scores of 3 (lowest score in our sample) on the Bem Sex Roles Inventory, while high femininity refers to individuals with scores of 7 (highest score in our sample) on the same inventory. Error bars represent standard errors of the mean. Asterisk (*) indicates significant findings (*p* < 0.05).

For stress symptoms, the analysis yielded a main effect of sex [*F*_(1, 140)_ = 12.09, CI_95%_ = 1.67 to 6.23, *p* = 0.001] and a trend for time [*F*_(1, 386)_ = 2.99, CI_95%_ = −0.05 to 0.80, *p* = 0.085]. No main effect of femininity [*F*_(1, 142)_ = 0.01, CI_95%_ = −1.57 to 1.48, *p* = 0.95] or masculinity [*F*_(1, 138)_ = −0.46, CI_95%_ = −1.78 to 0.86, *p* = 0.48] were found. The time^*^masculinity interaction reached significance [*F*_(1, 387)_ = 3.86, CI_95%_ = −1.93 to 0.12, *p* = 0.050] but *post-hoc* analyses revealed no significant effect. We also found a time^*^sex^*^femininity interaction [*F*_(1, 389)_ = 3.58, CI_95%_ = −2.54 to 0.06, *p* = 0.059], see Table 2. To decompose this three-way interaction as a function of femininity, comparisons of means were made with the scores of 3 (low) and 7 (high) on the Bem Sex Role Inventory scale as a function of time (T1 to T4) and sex on femininity scores. These scores were chosen based on the range of scores obtained in our sample (lowest score was 3 and highest score was 7). *Post-hoc* tests showed that females with high levels of femininity (7) had significantly (*p* ≤ 0.05) higher stress symptoms than males with high levels of femininity at T1, see Figure 2A.

**Table 2:** Linear mixed model to predict stress symptoms.

	**Main Effect**			**Interaction**		
	**Estimate**	**CI**	***P***	**Estimate**	**CI**	***p***
Intercept	11.00	0.86 to 21.15	0.031^*^	14.54	−5.19 to 34.27	0.140
Time	0.37	−0.05 to 0.80	0.084^†^	1.26	−5.86 to 8.39	0.725
Sex	3.95	1.67 to 6.23	<0.001^***^	−8.64	−33.02 to 15.74	0.477
Femininity	−0.04	−1.57 to 1.48	0.953	−0.77	−3.69 to 2.13	0.592
Masculinity	−0.46	1.78 to 0.86	0.482	−0.26	−3.18 to 2.65	0.857
Sex × Femininity				1.64	−2.01 to 5.28	0.367
Sex × Masculinity				0.62	−2.79 to 4.04	0.715
Time × Sex				4.48	−4.33 to 13.28	0.314
Time × Femininity				0.55	−0.47 to 1.57	0.285
Time × Masculinity				−0.90	−1.93 to 0.12	0.050^*^
Time × Sex × Femininity				−1.24	−2.54 to 0.06	0.059^†^
Time × Sex × Masculinity				0.62	−0.57 to 1.82	0.302
Marginal R^2^/Conditional R^2^			0.062/0.530			0.075/0.540

^***^p ≤ 0.001; ^**^p ≤ 0.01; ^*^p ≤ 0.05; ^†^*p* ≤ 0.10.

### 3.3. Main analyses for depression

For depressive symptoms, the analysis revealed a trend effect of time [*F*_(1, 384)_ = 3.11, CI_95%_ = −0.04 to 0.69, *p* = 0.079] but no effect of sex [*F*_(1, 139)_ = 1.61, CI_95%_ = −0.75 to 3.33, *p* = 0.213], femininity [*F*_(1, 141)_ = 0.21, CI_95%_ = −1.68 to 1.06, *p* = 0.655], or masculinity [*F*_(1, 137)_ = 2.61, CI_95%_ = −2.13 to 0.23, *p* = 0.113]. The analysis did not detect a significant interaction, see Table 3 and Figure 2B.

**Table 3:** Linear mixed model to predict depression symptoms.

	**Main effect**			**Interaction**		
	**Estimate**	**CI**	***p***	**Estimate**	**CI**	***p***
Intercept	11.45	2.37 to 20.53	0.012^*^	11.88	−5.64 to 29.39	0.175
Time	0.32	−0.04 to 0.69	0.078^†^	−1.41	−7.54 to 4.72	0.649
Sex	1.29	−0.75 to 3.33	0.206	0.79	−20.86 to 22.43	0.942
Femininity	−0.31	−1.68 to 1.06	0.650	0.10	−2.48 to 2.68	0.938
Masculinity	−0.95	−2.13 to 0.23	0.108	−1.52	−4.11 to 1.07	0.240
Sex × Femininity				−0.50	−3.73 to 2.74	0.759
Sex × Masculinity				0.69	−2.35 to 3.72	0.649
Time × Sex				2.95	−4.63 to 10.52	0.441
Time × Femininity				0.20	−0.68 to 1.08	0.655
Time × Masculinity				0.12	−0.76 to 1.01	0.784
Time × Sex × Femininity				−0.42	−1.54 to 0.70	0.460
Time × Sex × Masculinity				−0.10	−1.13 to 0.93	0.842
Marginal R^2^/Conditional R^2^			0.027/0.544			0.031/0.544

^***^p ≤ 0.001; ^**^p ≤ 0.01; ^*^p ≤ 0.05; ^†^*p* ≤ 0.10.

### 3.4. Main analyses for anxiety

For anxiety symptoms, results showed a main effect of sex [*F*_(1, 126)_ = 12.73, CI_95%_ = 1.21 to 4.32, *p* = 0.001] but no main effect of time [*F*_(1, 372)_ = 0.11, CI_95%_ = −1.37 to 12.49, *p* = 0.745], femininity [*F*_(1, 128)_ = 0.13, CI_95%_ = −1.23 to 0.85, *p* = 0.719], or masculinity [*F*_(1, 124)_ = 0.53, CI_95%_ = −1.23 to 0.57, *p* = 0.475]. We also found a time^*^sex^*^femininity interaction [*F*_(1, 376)_ = 3.79, CI_95%_ = −1.81 to 0.02, *p* = 0.052], see Table 4. Similar to the steps performed to decompose the three-way interaction as a function of femininity for stress symptoms, we used scores of 3 (low) and 7 (high) on the Bem Sex Role Inventory scale for anxiety symptoms. *Post-hoc* tests revealed that females with low levels of femininity had significantly (*p* ≤ 0.05) higher anxiety symptoms than males with low levels of femininity at T4, see Figure 2C.

**Table 4:** Linear mixed model to predict anxiety symptoms.

	**Main effect**			**Interaction**		
	**Estimate**	**CI**	***p***	**Estimate**	**CI**	***p***
Intercept	5.56	−1.37 to 12.49	0.109	10.41	−3.17 to 23.98	0.125
Time	0.05	−0.25 to 0.35	0.744	−3.43	−8.44 to 1.58	0.175
Sex	2.76	1.21 to 4.32	<0.001^***^	−3.96	−20.74 to 12.81	0.635
Femininity	−0.19	−1.23 to 0.85	0.715	−0.59	−2.59 to 1.42	0.557
Masculinity	−0.33	−1.23 to 0.57	0.467	−0.88	−2.89 to 1.12	0.376
Sex × Femininity				0.80	−1.71 to 3.30	0.525
Sex × Masculinity				0.46	−1.89 to 2.81	0.694
Time × Sex				5.13	−1.06 to 11.32	0.101
Time × Femininity				0.49	−0.23 to 1.21	0.178
Time × Masculinity				0.16	−0.56 to 0.88	0.664
Time × Sex × Femininity				−0.90	−1.81 to 0.02	0.052^†^
Time × Sex × Masculinity				0.01	−0.84 to 0.85	0.989
Marginal R^2^/Conditional R^2^			0.061/0.514			0.067/0.517

^***^p ≤ 0.001; ^**^p ≤ 0.01; ^*^p ≤ 0.05; ^†^ *p* ≤ 0.10.

## 4. Discussion

The main objective of this study was to examine the effect of sex and gender roles, as well as the interaction of sex and gender roles on symptoms of stress, depression, and anxiety during the COVID-19 pandemic. Over the course of this long-term stressful event, we found that females generally had more symptoms of stress and anxiety than males. We also found an interaction between femininity, sex, and time, such that females with high femininity had more stress symptoms at the beginning of the confinement measures compared to males with high femininity. Additionally, the results showed that females with low femininity had more anxiety symptoms one year after the confinement measures compared to males with low femininity. For depressive symptoms, no effect of gender roles or sex differences was found, nor any sex differences.

Contrary to our hypotheses, we did not find an association between masculine traits and symptoms of stress, anxiety, and depression. This result is surprising as it is inconsistent with previous studies showing that masculinity has a protective effect on mental health (Feather, 1985; Whitley, 1985; Nezu and Nezu, 1987; Stoppard and Paisley, 1987; Eisler et al., 1988; Grimmell and Stern, 1992; Kleinplatz et al., 1992; Thornton and Leo, 1992; Waelde et al., 1994; Bromberger and Matthews, 1996; Lengua and Stormshak, 2000; Stoyanova and Hope, 2012; Gibson et al., 2016; Arcand et al., 2020). One possible explanation for this finding is that the context of the pandemic may have prevented the positive attributes associated with masculinity to be used efficiently. The masculine scale of the questionnaire refers to characteristics of assertiveness and control. However, participants in our study were faced with a situation in which they had little control, had to remain confined to their homes, and had to respect a government-imposed curfew. Together, this may have dampened the expression of masculine traits. This provides insight into our lack of observed beneficial effects of masculinity on psychiatric symptoms. In addition, previous studies have reported that masculinity is associated with the use of active coping strategies and use of less avoidant coping strategies (Nezu and Nezu, 1987; Lengua and Stormshak, 2000). Although coping strategies were not measured in response to the pandemic in this study, individuals with high masculinity likely had coping styles (i.e., active) that could not be deployed as frequently as usual (prior to the pandemic) or that these coping styles did not present the usual beneficial effects. Taken together, our results suggest that masculinity does not appear to have a protective (or fragilizing) effect on psychiatric symptoms in the context of the COVID-19 pandemic. In addition, femininity influenced stress and anxiety symptoms but appeared to be a context-specific effect of the pandemic.

Indeed, our results show that females with more feminine traits were more stressed in the initial stages of the confinement measures compared to males with high feminine traits. The very few studies that have explored the impact of psychological gender roles on stress symptoms have reported a negative association between feminine traits and stress symptoms (Kleinplatz et al., 1992; Littlefield, 2004). However, these studies measured gender roles categorically, which could explain the discrepancy between the literature and results from our study. Sarrasin et al. (2014) reported that femininity was positively associated with threat perception in females, whereas this result was not found in males. Additionally, it has been shown that males tend to perceive stressful situations as challenges compared to females. However, it is unknown whether this effect is moderated by gender roles (Ptacek et al., 1992). In light of these findings, females with high levels of femininity may have interpreted the initial months of the pandemic as more threatening compared to males with high levels of femininity. Our results suggest that in the acute phase of pandemic-related stress, femininity seemed to have helped males and harmed females. To our knowledge, no study has investigated the impact of gender roles on stress symptoms in males. Importantly, some studies have reported that high femininity was negatively associated with mental health outcomes in males (Flett et al., 2009; Szpitalak and Prochwicz, 2013). In the current study, our results suggest that femininity did not increase stress symptoms in males. The typical negative effects of femininity may have been counteracted by the context of the pandemic, which may have been more adaptive for males exhibiting high levels of feminine traits. In fact, the social and economic shutdown triggered by the COVID-19 pandemic and public health measures in Quebec may have required males to suppress their typical masculine behaviors to favor more collectivistic behaviors that protect family and friends. Therefore, males may have appropriated more feminine gendered responsibilities to adapt to the pandemic. These results highlight the importance of contextualizing the use of gender roles, where different contexts could favor or oppose the adoption of given traits by each sex.

Our results also showed that females with few feminine traits were more anxious one year after the pandemic compared to males with few feminine traits. This surprising result is divergent from the existing literature that largely supports a positive association between femininity and anxiety symptoms (Palapattu et al., 2006; Blashill and Hughes, 2009; Arcand et al., 2020). In females, one study reported that femininity was positively associated with social support (Lengua and Stormshak, 2000), where the latter is known to be a protective factor against anxiety symptoms (Munir and Jackson, 1997). Our results align with this finding and suggest that low femininity may be especially harmful to females in the long run. Indeed, we only found an effect at T4, which corresponds to a year after the beginning of the implemented sanitary measures in Quebec. However, these finding warrants replication and is likely to be highly dependent on the contextual nature of the COVID-19 pandemic.

Interestingly, we only found effects for femininity in the current study. This result may be explained by the fact that our sample was more feminine than masculine. In addition, our study had twice as many females as males. For females, this finding is consistent with previous studies (Lengua and Stormshak, 2000; Johnson et al., 2006; Juster et al., 2016; Arcand et al., 2020), though this may be attributable to a sampling bias for males. As mentioned earlier, we re-contacted individuals who participated in our previous laboratory-based experiments to take part in this COVID-19 study. A large portion of our male sample (62%) stemmed from a previous study involving parent-child dyads. Studies have shown that gender roles can be modulated by different roles, worker/student status, and life experiences (Nezu and Nezu, 1987; Fan and Marini, 2000; Bryant, 2003; Kasen et al., 2006; Lemaster et al., 2017; Arcand et al., 2020). Therefore, it is possible that through the nature of their parental responsibilities, fathers exhibit more feminine traits than males without children. A larger sample would allow us to account for parental status in the statistical analyses.

Although we found sex differences for stress and anxiety symptoms, no differences were reported for depressive symptoms. In the literature, several mixed results have been reported. In general, females have higher depressive symptoms than males (Özdin and Bayrak Özdin, 2020; Xiong et al., 2020; Hyland et al., 2021), although a recent study found no sex differences for depressive symptoms (Shevlin et al., 2020). All while controlling for the number of days elapsed between data collection and March 2020, analyses of pre-pandemic depression scores were performed on our sample. Results showed that females were more depressed than males before the pandemic (*p* = 0.022), suggesting that the lack of sex differences observed in our COVID-19 study was likely due to symptoms of depression in response to the pandemic in males. In Quebec, confinement measures were drastic and resulted in the closure of all non-essential services (e.g., recreation, workplaces, schools). Consequently, individuals were forced to stay at home and limit activities outside of the household. As a result, this had a significant impact on factors that are closely related to depression, such as social connectedness (George et al., 1989).

In this study, we showed that individuals with greater psychological traits associated with femininity were better adapted to the COVID-19 pandemic. This suggests that the effect of gender roles on mental health symptoms is highly context dependent. Thus, some situations may favor individuals who possess more characteristics associated with femininity, while others may favor individuals with greater characteristics associated with masculinity.

This hypothesis supports the theory of androgyny proposed by Bem (1974) who argued that individuals with many feminine and masculine traits are able to better adapt to various situations. Thus, individuals with more rigid psychological gender roles (e.g., a male who identifies predominantly with feminine traits or a male who identifies predominantly with masculine traits) may have more difficulty adapting to situations that require them to display psychological traits that do not belong to their dominant gender role (Bem, 1981). Although these individuals may appear to be highly adapted in some situations, they may be less so in other contexts. Therefore, the development of both feminine and masculine traits should be encouraged in children and adolescents. This would allow youth to develop a larger toolbox that promotes their flexibility to adapt when exposed to different roles and contexts.

### 4.1. Limitations and future directions

Our results should be interpreted while considering certain limitations. First, every variable in our study stemmed from self-reports, including biological sex. The concept of gender is very broad and encompasses several subcategories including gender identity and gender relations (Johnson and Repta, 2012). As we focused on psychological gender roles in this study, this concept only addresses a portion of the multidimensional aspect of gender (Johnson and Repta, 2012). To adopt a more systemic approach, it would be relevant for future studies to include other aspects of gender (e.g., gender identity) to better understand the influence of the latter (Johnson and Repta, 2012). Further, although participants were all in good health at T0, we did not assess mental or physical health during the COVID-19 pandemic. With that said, studies tend to show that individuals with mental and physical health disorders may be more vulnerable to symptoms of stress, anxiety, and depression within the context of the pandemic (Özdin and Bayrak Özdin, 2020). Therefore, future studies should control for mental and physical health (healthy or disordered) to gain a better understanding of the impact of gender roles on symptom patterns during stressful events. Moreover, social support is known to moderate the relationship between femininity and depression. Given the current sample size and number of predictors in our statistical models, a lack of statistical power prevented us from including social support as a predictor in our model. Nevertheless, future studies should examine the contribution of social support to their findings given its moderating role in the relationship between femininity and mental health. In addition, it has been shown that males with depression tend to exhibit more externalizing symptoms than females (e.g., anger and aggression, substance abuse, and risk-taking; Martin et al., 2013). Therefore, depression scores quantified by the DASS may be underestimated in males. Future studies should utilize tools that are better adapted to the reality of males, such as the Male Depression Risk Scale (Rice et al., 2013). Finally, although we measured depression symptoms before the pandemic, we did not have data for stress and anxiety symptoms. It would be relevant for laboratories with baseline (pre-event) stress, anxiety, and depression data to re-measure these symptoms during a long-term stressful event to better understand the evolution of these symptoms over time.

## 5. Conclusion

Our findings revealed that females exhibited higher symptoms of stress and anxiety during the COVID-19 pandemic relative to males. Of note, we found that females with higher feminine traits had more stress symptoms at the beginning of the pandemic compared to males with higher feminine traits. However, females with less feminine traits were more anxious one year after the pandemic than males with less femininity. In this study, only feminine traits interacted with biological sex to predict stress and anxiety symptoms. As a result, our study supports the idea that the joint effect of sex and gender provides greater insight into our understanding of stress and anxiety symptoms in adults during a long-term stressful event such as the COVID-19 pandemic.

## Data availability statement

The raw data supporting the conclusions of this article will be made available by the authors, without undue reservation.

## Ethics statement

The studies involving human participants were reviewed and approved by Institutional Review Board of the CIUSSS-de-l'Est-de-l'Île-de-Montréal. The patients/participants provided their written informed consent to participate in this study.

## Author contributions

MA conducted the literature review, participated in the development of the study design, objectives of the article, performed the statistical analyses, and wrote the manuscript. AB-H helped with statistical analyses and created all graphics. R-PJ participated in the development of the study design and objectives of the article and revised the article. M-FM supervised all stages of the project and revised the article. All authors contributed to the article and approved the submitted version.
